# Coming of age: pharmacy practice in the 21st century

**DOI:** 10.1186/s13584-015-0058-z

**Published:** 2015-12-15

**Authors:** Donald F. Downing

**Affiliations:** University of Washington School of Pharmacy, 1959 NE Pacific Street, PO Box 357630, Seattle, WA 98105-7630 USA

## Abstract

Individuals, families, health plans and governments are struggling with the growing importance of managing chronic, non-communicable diseases. People in many countries are living longer and thus are facing many years of managing hypertension, diabetes and hyperlipidemia – often complicated by obesity, declining physical activity and changing diets. The financial burden this places on governments, employers, and individuals purchasing health care services is growing and solutions are being sought on how to both finance this care and deliver the best care possible. New ideas are emerging that look to newfound resources, and one untapped resource increasingly being utilized is the clinical pharmacist. After many years being primarily involved in medication supply-chain management, the assistance that highly skilled pharmacists can provide medical providers and their patients is now being recognized. In order to realize fully the benefits of clinical pharmacists, governments, health plans and medical providers are taking a second look at the wisdom of maintaining the long-standing policy of compensating pharmacists only for filling prescriptions.

## Background

The recent IJHPR article by Hila Yariv [1], “The Case of Pharmacist Prescribing Policy in Israel”, speaks well to the professional and policy difficulties encountered in finding reasonable solutions to increasing demands for access to quality health care services. Whereas access to medications may be the single biggest concern of most low and middle resource countries, countries of all resource levels are beginning to understand the need for better management of complicated chronic illnesses and the pressures this puts on provider and financial resources [2, 3]. The partial Israeli response to this pressure is referred to as pharmacy prescribing in Yariv’s article. This commentary looks at a much broader role that pharmacists can play in ameliorating unmet health care needs, not just by increasing access to more medications.

While medication use is often held to be essential to the mitigation of the human diseases, there are health policies, health care provider education standards, professional society policies, human behaviours, financial restraints, and environmental factors that prevent nations from realizing the full benefit that medications promise to provide. Much has been written that speaks to negotiating lower drug prices, restricting drug formularies, increasing patient cost-sharing, and broadening the use of generic drugs as key strategies to curbing drug costs. Yet, even with these tools in place, the goal of achieving improved health outcomes, while at the same time controlling drug costs, has been elusive. Well trained and well-meaning medical and pharmacy providers find themselves overwhelmed with managing the growing number of chronically ill patients, their prescription needs and their consultation needs. This commentary argues for reconsideration and repurposing of pharmacists as key to achieving the dual goals of improved health at an affordable cost that providers and governments seek. Success in this endeavour [4] arguably means setting aside long-held stereotypes of pharmacy practice and focusing policy more stridently on unmet patient needs and less on protection of traditional health care practices.

The 20th Century saw pharmacy practice come of age with the profession moving from a medication compounding specialty to the more expedient dispensing of drug manufacturers’ finished products. A vast array of powerful and effective medications became more available and pharmacists and pharmacies became an important supply chain component of that service. With finished products replacing the art of prescription compounding, pharmacists found that they were able to spend important time with their patients and they frequently became a first-stop trusted medication-use patient advisor and confidant. As time passed, however, pharmacists faced increasing demands to process more and more prescriptions and decreasing time to spend with patients. The lucrative business of dispensing large quantities of prescriptions generated significant revenues, and immense pharmacy chains developed, largely run by non-pharmacist shareholders. Drug manufacturers worked hard to influence medical providers to write more prescriptions for their products and influenced pharmacies to stock and dispense more of their products. As with pharmacists, medical providers found themselves pressured to see more patients in less time and financially were discouraged from spending as much time with their patients as they would have wanted. It was increasingly more expedient to send a patient out of the exam room with a new prescription than to take uncompensated time to coach patients on wellness-improving behaviours.

## The medical-pharmaceutical complex

This prescription pipeline, sometimes referred in part as a Medical-Pharmaceutical Complex, has influenced medical providers to prescribe more and newer medications and pharmacists to stock and dispense them. The prescription pipeline has served a number of purposes, not the least of which being the improved chances of drug manufacturers to see of a positive return on product development costs. Direct to consumer advertising (DTCA) by drug manufactures, where allowed, brings even more pressure to bear on medical and pharmacy providers to produce and fill more prescriptions. On November 17, 2015, the American Medical Association adopted policy that called for a ban on DTCA. According to AMA Board Chair-elect Patrice A. Harris, M.D., M.A., “Direct-to consumer advertising also inflates demand for new and more expensive drugs, even when these drugs may not be appropriate [5].” Drug formulary committees for health systems and health insurance plans are also heavily lobbied to add new and often more expensive medications to their formularies. While a medication access pipeline is important to patient care, proper drug therapy decisions must be evidence based and coordinated between all members of the health care team.

## Medication management

We would all agree that having access to essential medications is a good thing. This said, it must be also argued that while medication access is good, it is not nearly enough. Access must be to the most clinically appropriate medication, not just to medications that have been marketed most successfully [6]. Medications that are medically unnecessary; that are not sustainably affordable; that are dosed incorrectly; that are ineffective; that are not coordinated between providers; or that generate polypharmacy regimens that are too complex for patients to reasonably adhere to - all contribute to poor outcomes and rising healthcare costs. The mismanagement of medications is causative rather than preventative for hospitalizations, especially for the chronically ill elderly. Because many chronically ill patients see more than one prescribing medical provider, patients are also exposed to a host of potential drug-drug conflicts and fragmented drug therapy. Governments and health plans continually address policies that would lower the cost of prescriptions, but rarely do they speak to the cost savings associated with policies that incentivize improved clinical medication management. One only needs to look into the medicine cabinets of geriatric patients to visualize the ubiquity and seriousness of this issue.

In the United States, where marketplace incentives are historically preferred over government price controls, pharmacists are increasingly being used as medication consultants and health-team medication managers. Pharmacists act as consults for both patients and providers. They help reduce medication errors and preventable adverse drug events. Pharmacists are being invited into clinic primary care and specialty care teams to help medical providers manage greater numbers of complex therapy patients. In order to support these services in Washington State, an Attorney General’s informal opinion and recent legislation has confirmed that pharmacists must be contracted by health plans to provide post-diagnosis medication management and not just compensated for filling prescriptions [7].

For traditional dispensing pharmacies in the U.S., the fee-for-service incentives to fill prescriptions has been diminished by government and private health plans who have reduced prescription dispensing fees in their efforts lower drug expenditures [8]. As a consequence, however, pharmacists find it increasing difficult to justify providing free clinical care and enough time with patients to help them sort out their medication issues. In fact, surveys show that reduced interaction time between a patient and a pharmacist has altered patient perspectives as to what a desirable pharmacy visit entails. In most cases patients express the opinion that a good pharmacy visit provides fast service, low cost drugs, and in some countries the provision of stay-in-your-automobile, drive-through lanes or mail-order drug delivery. In low resource countries most medications aren’t dispensed by pharmacists at all. While these creative drug pipe-line innovations may be convenient to busy patients they are not likely to be conducive to well-managed medication use and decreased health costs.

## Aligning incentives

Pharmacists graduating from colleges with clinical doctorates rightfully question why they are only compensated for filling prescriptions when their professional education is based on medication management services. These pharmacists are exquisitely suited and professionally obligated to work with patients and their medical providers to make sure that patients are only taking the medications that are clinically appropriate – often leading to a decision to reduce the patient’s prescription load. Reducing a patient’s prescription burden, however, reduces a pharmacist’s income. The integrity of pharmacist decision-making is compromised by this long-standing health policy model and puts patients at greater financial and clinical risk. For governments who have and support public universities, their dollars used to support pharmacist education seem arguably misspent if that government’s health policies only incentivize their pharmacy school graduates to sell more and more medications rather than using these experts to work with medical providers to make sure limited resources are spent on the most clinically and fiscally appropriate medications.

## A few models of pharmacy services

There are many models of patient-centric health care teams that include pharmacists as core team members. Many pharmacists, rather than dispensing medications, have patients who are referred to them after a diagnosis by a medical provider. Patients with chronic illnesses can be managed by pharmacists after the diagnosis has been made and thus free up medical providers to see the more acutely ill. Classically, pharmacists who run anticoagulation management services for medical clinics, find significantly more patients within INR goals and with fewer bleeding or clotting mishaps and hospitalizations [9]. Not only does this service result in better clinical and financial outcomes, it frees medical providers for other care services. Pharmacists may be employed as a member of a medical clinic’s clinical team or contracted externally. To be clear, medical providers still control final prescribing decisions and pharmacists are not competing for medical provider patients. Billable pharmacist services for medication management most commonly are predicated on formal referrals or on previously authorized written protocols for prevention-based services such as immunizations.

## Conclusion

As governments, private health plans and patients struggle with the financial burdens of managing chronic illnesses, new and innovative methods of care delivery need to be considered. The approach described in the commentary has gained significant momentum and makes use of an existing underutilized resource – clinical pharmacists. It does require a progressive and inter-professional program of pharmacy/medical education, greater coordination of care, and health policies that incentivize all members of the health care team to provide collaborative primary care. Pharmacy graduates with requisite clinical training are eager and able to help solve the time and financial constraints facing primary care providers trying to manage the unabated number of individuals who are chronically ill.

While one might expect strident medical opposition to these pharmacist services, I have been told by physicians who have practiced under these circumstances that they wish that this arrangement had been established earlier. Not only have they found that their work-life improved with a pharmacist on their team, but they also expect improvements in the health outcomes of their chronically ill patients. This benefits not only the patients but those physicians for whom improved quality of care and outcomes lead to improved medical provider payments rather than revenue reductions.
